# Bench experiments comparing simulated inspiratory effort when breathing helium-oxygen mixtures to that during positive pressure support with air

**DOI:** 10.1186/1471-2466-12-62

**Published:** 2012-10-03

**Authors:** Andrew R Martin, Ira M Katz, Katharina Jenöfi, Georges Caillibotte, Laurent Brochard, Joëlle Texereau

**Affiliations:** 1 Medical Gases Group, Air Liquide Santé International, Centre de Recherche Claude-Delorme, Jouy-en-Josas, France; 2 Department of Mechanical Engineering, Lafayette College, Easton, PA, USA; 3 Medical Intensive Care Unit, University Hospital, University of Geneva, Geneva, Switzerland; 4 Current affiliation: Medical Gases, Delaware Research and Technology Center, American Air Liquide, Newark, DE, USA

**Keywords:** Helium, Oxygen, Heliox, Inspiratory effort, Work of breathing, Airway resistance, Lung compliance, Non-invasive ventilation, Pressure support

## Abstract

**Background:**

Inhalation of helium-oxygen (He/O_2_) mixtures has been explored as a means to lower the work of breathing of patients with obstructive lung disease. Non-invasive ventilation (NIV) with positive pressure support is also used for this purpose. The bench experiments presented herein were conducted in order to compare simulated patient inspiratory effort breathing He/O_2_ with that breathing medical air, with or without pressure support, across a range of adult, obstructive disease patterns.

**Methods:**

Patient breathing was simulated using a dual-chamber mechanical test lung, with the breathing compartment connected to an ICU ventilator operated in NIV mode with medical air or He/O_2_ (78/22 or 65/35%). Parabolic or linear resistances were inserted at the inlet to the breathing chamber. Breathing chamber compliance was also varied. The inspiratory effort was assessed for the different gas mixtures, for three breathing patterns, with zero pressure support (simulating unassisted spontaneous breathing), and with varying levels of pressure support.

**Results:**

Inspiratory effort increased with increasing resistance and decreasing compliance. At a fixed resistance and compliance, inspiratory effort increased with increasing minute ventilation, and decreased with increasing pressure support. For parabolic resistors, inspiratory effort was lower for He/O_2_ mixtures than for air, whereas little difference was measured for nominally linear resistance. Relatively small differences in inspiratory effort were measured between the two He/O_2_ mixtures. Used in combination, reductions in inspiratory effort provided by He/O_2_ and pressure support were additive.

**Conclusions:**

The reduction in inspiratory effort afforded by breathing He/O_2_ is strongly dependent on the severity and type of airway obstruction. Varying helium concentration between 78% and 65% has small impact on inspiratory effort, while combining He/O_2_ with pressure support provides an additive reduction in inspiratory effort. In addition, breathing He/O_2_ alone may provide an alternative to pressure support in circumstances where NIV is not available or poorly tolerated.

## Background

The effects of inhaling helium-oxygen mixtures (He/O_2_) during spontaneous breathing continue to be explored for the treatment of obstructive lung diseases, both during acute exacerbations of disease [1-7] and during the exercise component of rehabilitation programs [8-11]. When breathing He/O_2_, the low density of the mixture compared to air reduces airway resistance, specifically, density-dependant components of airway resistance that arise from turbulent flow, and from convective acceleration and deceleration of gas as it passes through the branching network of the tracheobronchial airways [12-14]. For patients suffering from obstructive lung diseases, the benefits of decreased airway resistance are many, including alleviation of dyspnea [15], reduced expiratory time constants leading to improved operational lung volumes [11,16], and a reduced work of breathing [13]. However, intersubject variability in response to breathing He/O_2_ is large [3,12,13], resulting at least in part from variation in morphological phenotypes of disease between patients. Such variability has likely contributed to inconclusive results regarding the clinical effectiveness of He/O_2_ in studies performed to date [1,2,6].

In many of the same applications for which He/O_2_ is being explored, non-invasive ventilation (NIV) is already widely employed. Positive pressure support delivered via NIV may be used to unload the respiratory muscles, as a portion of the overall work of breathing is performed by the ventilator and not by the patient. At a sufficiently high level of pressure support, the effort required of the patient is as little as that necessary to trigger cycling between inspiratory and expiratory phases of ventilation, allowing the respiratory muscles to unload. In many circumstances, however, pressure support may not be well tolerated by the patient. Moreover, higher levels of pressure support increase the occurrence of leaks at the patient-mask interface, which play a major role in generating patient-ventilator asynchrony [17]. The combination of pressure support NIV with He/O_2_ has been explored under the hypothesis that individual benefits of the two therapies are additive [13,15,18]. Indeed, during exacerbations of chronic obstructive pulmonary disease, pressure support with He/O_2_ has been shown to reduce dyspnea [15] and work of breathing [13] more so than pressure support with air/O_2_. It may therefore follow that patient outcomes can be improved at lower levels of pressure support for He/O_2_ than for air/O_2_. Taken further, for those patients that respond strongly to He/O_2_, breathing He/O_2_ alone may decrease the work of breathing sufficiently to achieve clinical improvement without simultaneous pressure support, making He/O_2_ a viable alternative for patients that do not tolerate pressure support NIV, or in circumstances in which NIV is not readily available.

The present work was conducted in order to explore the effects of He/O_2_ and of positive pressure support, both separately and in combination, on inspiratory effort simulated using a mechanical test lung. Measurements were taken for different breathing patterns, for different concentrations of helium and oxygen, and across a range of simulated adult, obstructive disease patterns, in an effort to better identify factors influencing variation in patient response to He/O_2_.

## Methods

### Experimental apparatus

**Figure 1:**
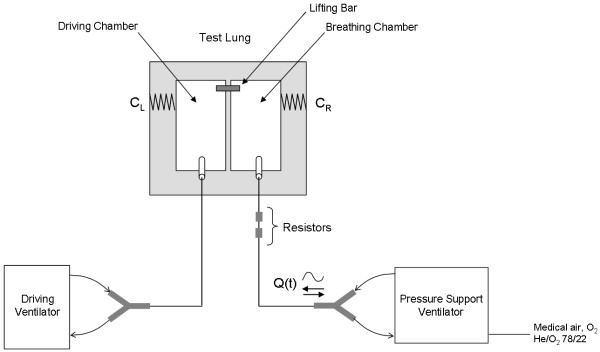
**Schematic diagram of the experimental apparatus.** Q(t) is the flow rate into and out of the breathing chamber as a function of time, and C_L_ and C_R_ are the compliances of the left (driving) and right (breathing) chamber, respectively.

The experimental apparatus is displayed schematically in Figure 1. Patient breathing was simulated using a dual chamber adult test lung (Michigan Instruments, USA) with the two chambers connected via a lifting bar, and the driving chamber connected to a ventilator (Neftis ICU; Taema, France, or Monnal T75; Air Liquide Medical Systems, France) operated in volume control mode to impose breathing patterns. The second chamber, referred to henceforth as the breathing chamber, was either left open to room air or connected to a Hamilton G5 ventilator (Hamilton Medical AG, Switzerland) operated in NIV mode with medical air or He/O_2_ (at either 78% He, 22% O_2_ or 65% He, 35% O_2_).

### Simulated breathing and ventilatory support

Experiments were performed for each gas mixture with a pressure support of 0 cm H_2_O and positive end-expiratory pressure (PEEP) of 0 cm H_2_O, as well as with pressure support levels of 10 and 15 cm H_2_O, both above PEEP of 5 cm H_2_O. In all of these cases, the ventilator was operated with a pressure trigger of -2 cm H_2_O. Three adult breathing patterns were simulated, each with a square wave inspiratory flow pattern, and an inspiratory/expiratory ratio of 1/2: the first had a tidal volume of 500 mL and a breathing frequency of 15 breaths/min, producing an average inspiratory flow rate of 22.5 L/min, the second had a tidal volume of 600 mL and a breathing frequency of 20 breaths/min, producing an average inspiratory flow rate of 36 L/min, and the third had a tidal volume of 700 mL and a breathing frequency of 25 breaths/min, producing an average inspiratory flow rate of 52.5 L/min. Tidal volumes were set at the driving ventilator and monitored with the pressure support ventilator. At the start of each series of measurements, the flow sensor of the pressure support ventilator was calibrated for medical air or the He/O_2_ mixture used following the normal operating procedures for the ventilator. Where a discrepancy was observed between the set tidal volume and the monitored tidal volume, as occurred for high operating pressures, the setting at the driving ventilator was adjusted to produce the desired tidal volume as measured at the pressure support ventilator. For experiments performed with room air and no pressure support ventilator, set tidal volumes were monitored and adjusted as necessary according to the graduated scale provided on the test lung.

### Resistance and compliance settings

Combinations of parabolic airway resistors (Rp5 and/or Rp20) were inserted in series at the inlet to the breathing chamber, separated by a 42 cm length of 12 mm ID tubing provided with the test lung, to simulate different levels of airway resistance. These resistors obey the relationship $\Delta P=\frac{k}{2}\rho {\bar{U}}^{2}$, where *ΔP* is the pressure loss across the resistor, *ρ* is the gas density, *Ū* is the gas velocity averaged over the cross-section of the resistor, and k is a constant that, over the conditions tested, is a function of only the geometry of the resistor. That is to say, k is a useful parameter, borrowed from the fluid mechanics literature [14,19,20], through which to express the severity of the density-dependant component of airway resistance independently from gas composition. In what follows, we will refer to the parameter k as the resistive loss coefficient. For reference, equivalent values of the linear resistance, R, are provided in Table 1 for air and He/O_2_ 78/22 for different combinations of parabolic resistors, and at the three flow rates studied. At a fixed resistive loss coefficient, the simulated inspiratory effort was assessed with breathing chamber compliances of 0.02, 0.05, and 0.10 L/cm H_2_O by adjusting a spring according to the graduated scale on the test lung. In addition to the parabolic resistors, further experiments were performed with two breathing filters (Clear-Guard 3; Intersurgical, UK), each with a nominally linear resistance of 2 cm H_2_O L^-1^ s, stacked in series at the inlet to the breathing chamber. In this case, the test lung tubing was removed so as to minimize density-dependent, nonlinear resistance. Prior to performing experiments with the test lung, the pressure drop across these two filters, including connections to the breathing chamber and the support ventilator circuit, was measured using a digital manometer (PR-201; Eurolec, Ireland) over a range of known, steady flow rates of medical air or He/O_2_ 78/22 supplied by a mass flow controller (EL-FLOW Select; Bronkhorst High-Tech, Netherlands).

**Table 1:** Resistive loss coefficients (k) and equivalent linear resistances for air and helium/oxygen 78/22 for combinations of parabolic resistors used with the test lung

			**R [cm H**_**2**_**0 L**^**-1**^**s]**	
	**k**	**Q[L/min]**	**Air**	**He/O**_**2**_**78/22**
Rp5	3.3	22.5	1.0	0.4
		36.0	1.6	0.6
		52.5	2.4	0.8
Rp20	21.5	22.5	6.6	2.3
		36.0	10.6	3.7
		52.5	15.4	5.4
2xRp5	6.6	22.5	2.0	0.7
		36.0	3.2	1.1
		52.5	4.7	1.7
Rp5 + Rp20	24.8	22.5	7.6	2.7
		36.0	12.2	4.3
		52.5	17.8	6.2
2xRp20	43.0	22.5	13.2	4.6
		36.0	21.1	7.4
		52.5	30.8	10.8

k is defined in the relationship $\Delta P=\frac{k}{2}\rho {\bar{U}}^{2}$, where *ΔP* is the pressure loss across the resistor, *ρ* is the gas density, and *Ū* is the mean gas velocity entering the resistor.

### Determination of inspiratory effort

For each experiment, pressure versus time and flow versus time data were recorded from the driving ventilator. The total work done by the driving ventilator on the system was then calculated by numerically integrating the product of pressure and flow with respect to time. In order to estimate the simulated inspiratory effort from the total calculated work, two corrections were applied to subtract work done on the driving side of the test lung. First, the work done to lift the driving chamber itself, as determined from preliminary experiments performed at equivalent breathing patterns, but without the lifting bar in place (that is, without the additional load of the breathing chamber), was subtracted from the total work. Second, flow rates recorded from the driving ventilator were scaled by the ratio between the tidal volume measured by the support ventilator and that reported by the driving ventilator. For experiments performed with room air, flow rates were instead scaled according to the tidal volume observed for the breathing chamber according to the graduated volume scale provided on the test lung. This correction was made to account for additional work done on the driving side of the test lung at high pressures, owing to the compliance of tubing connecting the driving ventilator to the driving chamber, and to horizontal expansion of the driving chamber bellows (i.e., where the intended expansion is in the vertical direction to lift the top plates of the test lung). If ever these two corrections resulted in a negative estimated inspiratory effort, the negative value was deemed physiologically unrealistic, and a value of zero was instead assigned. Clearly, this assignment neglects patient effort required to trigger the support ventilator; however, it will be argued below that over the range of parameters studied, this effort was small. All experiments were repeated a minimum of two times in order to estimate the measurement precision.

## Results

With all parameters held constant, the inspiratory effort measured for medical air supplied through the support ventilator with zero pressure support and zero end-expiratory pressure was only slightly greater than that for room air, the difference ranging from 0.05 J/L to 0.15 J/L from lowest to highest resistance studied. Accordingly, the work imposed by the ventilator was judged sufficiently small that data obtained at zero pressure support is representative of unassisted spontaneous breathing.

**Figure 2:**
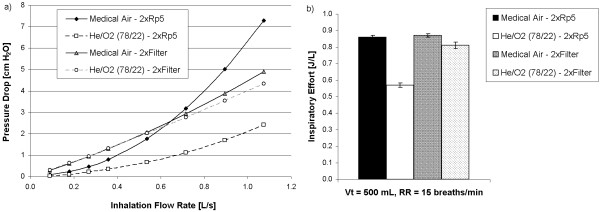
**a) The relationship between pressure drop and flow rate, and b) the inspiratory effort during simulated unassisted spontaneous breathing at a fixed tidal volume (V**_**t**_**) and respiratory rate (RR) are compared between non-linear (2xRp5) and nominally linear (2xFilter) airway resistances for medical air and for helium/oxygen 78/22.**

Figure 2a displays the relationship between pressure drop and flow rate of medical air or He/O_2_ (78/22) for the stack of two breathing filters, which can be viewed as a nominally linear resistance. For comparison, pressure drop measurements made for the combination of two Rp5 resistors connected with the test lung tubing are also shown. Figure 2b gives the inspiratory effort measured at a fixed breathing pattern for medical air and He/O_2_ (78/22), with the same resistances placed in the breathing side of the test lung.

**Figure 3:**
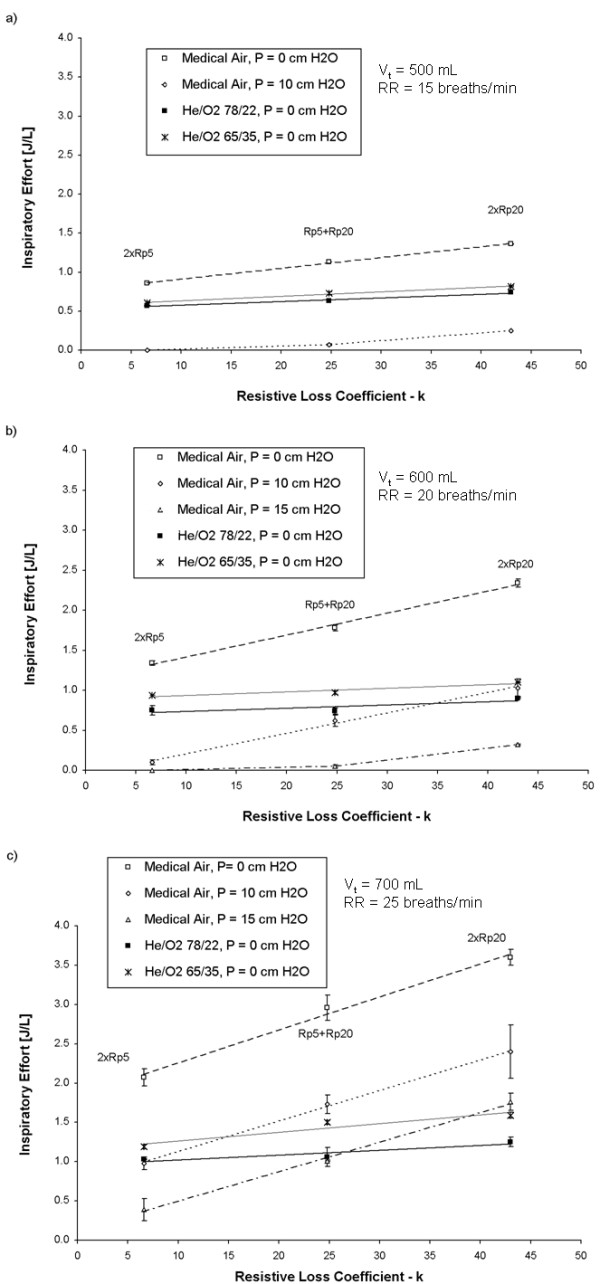
**Inspiratory effort during simulated unassisted spontaneous breathing (P = 0 cm H**_**2**_**O) is plotted versus the resistive loss coefficient, with compliance held constant at 0.05 L/cm H**_**2**_**O, for medical air, and two helium/oxygen mixture (78/22 or 65/35) at a) a respiratory rate (RR) of 15 breaths/min, and a tidal volume (V**_**t**_**) of 500 mL, b) a respiratory rate (RR) of 20 breaths/min, and a tidal volume (V**_**t**_**) of 600 mL, and c) a respiratory rate (RR) of 25 breaths/min, and a tidal volume (V**_**t**_**) of 700 mL.** For medical air, data is also shown for positive pressure support of 10 or 15 cm H_2_O, supplied with a positive end-expiratory pressure of 5 cm H_2_O. Error bars represent standard deviations around mean values.

**Figure 4:**
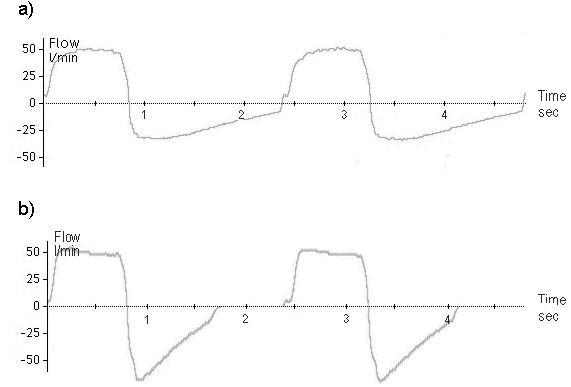
**Example flow versus time curves measured over two breathing cycles by the pressure support ventilator for a) medical air and b) helium/oxygen 78/22supplied with zero pressure support.** Curves were obtained for a breathing chamber compliance of 0.05 L/cm H_2_O, a resistive loss coefficient of 43.0, a respiratory rate of 25 breaths per minute, and a tidal volume of 700 ml.

Figure 3 compares the relative influences of the resistive loss coefficient, level of pressure support, administered gas, and breathing pattern on inspiratory effort. In addition, Figure 4 displays example flow versus time curves for medical air and He/O_2_ (78/22) as measured by the pressure support ventilator. The breathing chamber compliance was held constant at 0.05 L/cm H_2_O during these experiments. For medical air, inspiratory effort increased in a consistent manner with increasing resistive loss coefficient, decreased with increasing pressure support, and, all else being equal, was greater for breathing patterns producing higher inspiratory flow rates (Figure 3). For He/O_2_, increasing resistance had much less impact on inspiratory effort than for air, such that the reduction in inspiratory effort measured for He/O_2_ was greater for larger resistive loss coefficients. The effect of changing the mixture concentration of He/O_2_ from 78/22 to 65/35 was relatively small compared with the effects of other parameters studied. Results for He/O_2_ supplied with a pressure support of 10 or 15 cm H_2_O are not shown in Figure 3, as over the range of parameters presented in the figure the measured inspiratory effort was either zero or very small.

**Figure 5:**
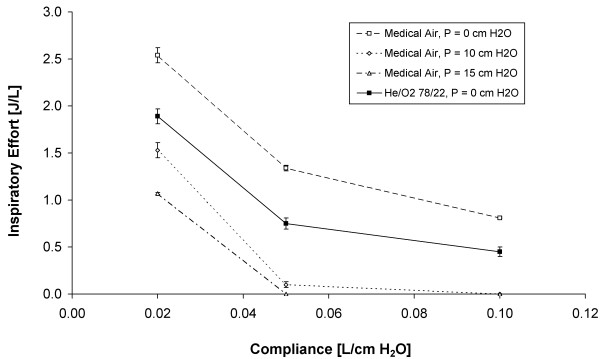
**Inspiratory effort breathing medical air or helium/oxygen 78/22 versus the breathing chamber compliance, with the resistive loss coefficient held constant at k = 6.6 (corresponding to two Rp5 resistors in series), a respiratory rate of 20 breaths/ min, and a tidal volume of 600 mL.** Positive pressure support of 10 or 15 cm H_2_O was supplied with a positive end-expiratory pressure of 5 cm H_2_O. Error bars represent standard deviations around mean values.

Figure 5 displays measured values of inspiratory effort with the breathing pattern and airway resistance held constant, and with breathing chamber compliances of 0.02, 0.05, and 0.10 L/cm H_2_O. For medical air inspiratory effort decreased with increasing pressure support. In addition, as compliance decreased, inspiratory effort increased nonlinearly regardless of the gas mixture. Results for He/O_2_ supplied with a pressure support of 10 or 15 cm H_2_O are not shown in Figure 5, as inspiratory effort was zero at either support level for compliances of 0.05 or 0.10 L/cm H_2_O.

In contrast, at a compliance of 0.02 L/cm H_2_O, considerable levels of inspiratory effort were measured for all conditions studied, including for the combination of positive pressure support with He/O_2_. These data are displayed in Table 2 for the 25 breaths/min, 700 mL tidal volume breathing pattern, along with the work done by the ventilator providing pressure support, determined as the difference between inspiratory effort measured at zero pressure support and those measured with support of 10 or 15 cm H_2_O. Theoretical values of the work done by the ventilator are also included in Table 2. These were calculated under the idealized assumption of an instantaneous rise in pressure at the start of inhalation, so that the support pressure was considered constant over the entire inhaled volume.

**Table 2:** **Inspiratory effort for varying levels of pressure support for C = 0.02 L/cm H**_**2**_**O, k =6.6, V**_**t**_**=700 ml, and RR = 25 bpm**

			**Ventilator Work [J/I]**	
**Gas**	**P**_**support**_**[cm H**_**2**_**0]**	**Inspiratory Effort [J/I]**	**Theoretical**	Experimental*
Medical Air	0	3.52 ± 0.25		
	10	2.66 ± 0.11	1.00	0.86 ± 0.36
	15	2.08 ± 0.03	1.50	1.44 ± 0.28
He/O_2_78/22	0	2.45 ± 0.01		
	10	1.77 ± 0.20	1.00	0.68 ± 0.21
	15	1.15 ± 0.01	1.50	1.30 ± 0.02

*Determined from the difference between inspiratory effort at P_support_ = 0 and that at P_support_ = 10 or 15 cm H_2_O.

## Discussion

### Interpretation of experimental results

The experiments presented above were conducted using a mechanical test lung with the goal of comparing the effects of He/O_2_ breathing and positive pressure support on simulated inspiratory effort across a range of obstructive phenotypes. The test lung provides a physical, conceptual model of the human respiratory tract. Clearly, some salient features of the respiratory system are not present in the test lung, so that potential effects on inspiratory effort of, for example, changes in ventilation distribution, dynamic airway closure, or respiratory muscle recruitment were not modeled in the present work. That said, the use of a mechanical test system has enabled us to perform controlled, parametric experiments which we’ve found useful in examining the manner in which He/O_2_ and pressure support affect inspiratory effort. While it should be noted that increased resistance in obstructive lung diseases may also markedly impact expiration, the work presented primarily addresses effects on inspiratory effort, as reduction of a patient’s inspiratory effort and work of breathing is a main goal of ventilatory support.

Positive pressure support was supplied to the breathing chamber using an ICU ventilator operated in NIV mode. While leaks at patient interfaces clearly influence ventilator performance during NIV [14], these particular experiments were performed under the ideal condition of no leak, in order to eliminate effects of ventilator-specific leak compensation algorithms and patient-ventilator asynchrony to focus instead on the more fundamental influence of gas properties on lung mechanics.

The mechanical work spent during inhalation consists of an elastic component required to expand the lung and chest wall, and a resistive component required to move gas through the airways. Additional components of work resulting from non-elastic deformation of tissues are comparably small [21]. In the present experiments, elastic work was performed as the breathing chamber of the test lung expanded against the force of its spring, the position of which was adjusted in order to simulate varying levels of compliance. Resistive work was associated with the passage of gas through the airway resistors. Such resistors are used to represent pressure losses at varying flow rates through the complex network of airways making up the respiratory tract. The pressure loss across the parabolic resistors predominantly used in this study is dominated by inertial effects, so that it varies with gas density and with the square of gas velocity. In contrast, the nearly linear resistance presented by the breathing filters (Figure 2a) arises mainly due to effects of gas viscosity during passage through the porous filter medium, with gas density playing only a minor role due to the persistence of small inertial effects. In general, parabolic resistors may be thought to represent pressure losses that arise from obstructions occurring in upper airways and the larger airways of the lung, where pressure losses are also inertial in nature, whereas linear resistors are representative of the smaller, peripheral airways, in which flow is sufficiently slow that viscous pressure losses dominate. The density-dependence of inertial pressure losses has been widely attributed to the presence of turbulence in the upper airways and bronchi; however, several authors have noted that even under laminar flow conditions pressure losses may maintain an inertial dependence due to convective acceleration occurring as flow changes direction, for example, at airway bifurcations [12-14,22,23]. The latter point is important in understanding the manner in which breathing He/O_2_ influences airway resistance and work of breathing, given that the magnitude of inertial pressure losses decreases with decreasing gas density. Indeed, a recent numerical analysis demonstrated that at elevated inspiratory flow rates He/O_2_ is expected to reduce pressure losses (i.e. airway resistance) down to approximately the 10^th^ lung generation, where flow is laminar [14].

With the above in mind, it should be no surprise that the reduction in inspiratory effort observed in the present experiments for He/O_2_ compared to air depended strongly on the magnitude and type of simulated airway obstruction. As observed in Figure 2b, the effect of He/O_2_ on inspiratory effort was considerably smaller for the breathing filters than for the parabolic resistors. During these experiments, the inspiratory flow waveform observed at the support ventilator rose sharply to approximately 35 L/min (~0.6 L/s) at the start of inhalation, and then flattened to a value of approaching 20 L/min (~0.33 L/s). Referring to Figure 2a, over these flow rates there is a considerable reduction in the pressure drop across the parabolic resistors for He/O_2_ compared to air, whereas the pressure drop across the filters is essentially equal for the two gases. Accordingly, the contrasting influence of He/O_2_ on inspiratory effort seen in Figure 2b can primarily be attributed to the different flow-pressure drop relationship for the two types of resistance.

In Figure 3, inspiratory effort is plotted against the resistive loss coefficient defined earlier (see Table 1) for parabolic resistors. For air, the application of pressure support of either 10 or 15 cm H_2_O decreased inspiratory effort in a consistent manner, independent of the resistive loss coefficient, below that measured for zero support. When no pressure support was provided, the simulated inspiratory effort was equivalent to the total work required for inspiration. With the application of pressure support, this total work was partitioned between the inspiratory effort and work done by the ventilator providing support. That is to say, pressure support lowered inspiratory effort by performing a portion, or in some cases nearly all, of the total work of inspiration. In contrast, substituting He/O_2_ for air decreased the resistive component of the total work of inspiration. As a consequence, even without pressure support, the inspiratory effort was reduced. Only small differences in inspiratory effort were measured between the two He/O_2_ mixtures (78/22 and 65/35), consistent with the relatively minor differences in gas properties between these two mixture concentrations [24], and suggesting that varying the O_2_ concentration within this range would have little influence on the clinical efficacy of He/O_2_ mixtures.

Whereas the compliance of the breathing chamber was fixed throughout the experiments summarized in Figure 3, Figure 5 demonstrates the influence of varying compliance on inspiratory effort. The inspiratory effort rose sharply as compliance decreased, regardless of whether air or He/O_2_ was inhaled. The reduction in inspiratory effort between air and He/O_2_ was constant for a given breathing pattern because in conducting this set of experiments only two Rp5 resistors were used (so that the resistive loss coefficient was held constant). Accordingly, it can be concluded that, in the present experiments, He/O_2_ affected only the resistive component of the inspiratory work. It is important to note that although He/O_2_ has no effect on the elastic work of breathing for the mechanical test lung, significant effects might be expected for patients experiencing expiratory flow limitations and associated dynamic hyperinflation. For these patients, the elastic work of breathing is elevated because lung compliance decreases as lung volume increases towards total lung capacity (TLC) [25]. He/O_2_ can improve expiratory flow by lowering airway resistance [26-28], so as to reduce end-expiratory lung volumes and improve compliance, which in turn will decrease the elastic work of breathing upon inhalation. In the present experiments, replacing air with He/O_2_ allowed the breathing chamber to empty faster (Figure 4); however, this did not translate to reduced elastic work because the volume to which the test lung chamber was inflated had little influence on its compliance. Moreover, while the expiratory flow curves in Figure 4 indicate that ventilation with He/O_2_ eliminated end-expiratory gas trapping that occurred with air for the case shown, in the majority of cases studied, no end-expiratory gas trapping developed, neither for air nor He/O_2_.

In addition to lowering end-expiratory lung volumes as described above, improved expiratory flow when breathing He/O_2_ may also lower the corresponding intrinsic PEEP in the lung. When the intrinsic PEEP within the lung is greater than the PEEP supplied at the level of the ventilator, the patient must do added work to lower the pressure at the ventilator by an amount sufficient to trigger inspiratory pressure support. In such circumstances, breathing He/O_2_ may be expected to reduce the triggering work by decreasing or eliminating intrinsic PEEP. In the present work, such effects likely occurred for the most obstructive cases; however, as intrinsic PEEP was not directly measured, this component of the work of breathing was not quantified explicitly.

Figures 3 and 5 do not include data for the combination of He/O_2_ with pressure support, as in the majority of cases studied this resulted in near zero inspiratory effort. Experiments at low breathing chamber compliance, for which significant amounts of inspiratory effort were measured for He/O_2_ combined with pressure support, are summarized in Table 2. As these experiments were conducted with a fixed level of resistance, the absolute difference in inspiratory effort between air and He/O_2_ was the same (within experimental error) for each level of pressure support. Concurrent to this difference in resistive work, the effect of pressure support on inspiratory effort was the same for He/O_2_ as for air: a portion of the total work required for inspiration was performed by the support ventilator, leaving less work for the driving ventilator (the patient) to perform. It is not surprising that experimental values of the ventilator work were below the theoretical values, given that the latter were calculated assuming an instantaneous rise in pressure at the start of inhalation, whereas in the experiments there was a small lag between the initiation of a new breath and ramp-up of pressure support. The results presented in Table 2 provide strong evidence that He/O_2_ and pressure support may be used in a complementary, additive manner to lower the work of breathing for patients with severe airway obstruction. This conclusion is well aligned with the results of Jaber and colleagues [13] for a small group of patients suffering acute exacerbations of COPD, where use of He/O_2_ during pressure support NIV enhanced the reduction in patient effort provided by NIV with air/O_2_ mixtures.

### Implications for clinical investigation of helium/oxygen mixtures

The experiments presented above were conducted in part to gain insight into variation in patient response to He/O_2_. Though the use of He/O_2_ in respiratory medicine has been a subject of considerable research for many decades, widespread clinical use has yet to follow. Previously, the lack of delivery devices specifically designed for use with He/O_2_ has impeded its adoption [29-31]. The recent development of He/O_2_-compatable devices, including the ventilator employed in the present study, aims to remove this obstacle. However, the fact remains that patient response to breathing He/O_2_ is highly variable [3,12,13]. It is clear that the direct effects of He/O_2_ on reducing airway resistance and the resistive work of breathing, and indirect effects on elastic work due to improved expiratory flow, depend strongly on the severity and location of airway obstruction [12,32]. The latter, the location of obstruction, is especially important in determining the manner in which pressure loss through an obstructed airway varies with gas flow rate and density. More than fifty years ago, building on foundational work on airway resistance by Rohrer [33], Arthur Otis and colleagues [21,23,34,35] were careful to separate airway resistance into two components: a viscous resistance for which pressure loss varies linearly with flow rate, and a second component, resulting from convective acceleration of flow (e.g. at bifurcations) [23] and/or turbulence [21,23], for which the pressure loss varies with the square of the flow rate, and also with gas density. Further, these researchers described experimental procedures through which the magnitude of each of these components could be estimated for a specific individual [21,23,34]. Today, the relationship between pressure loss and flow through airways is commonly approximated as linear, so that resistance may be described by a single parameter (expressed in cm H_2_O L^-1^ sec). Though convenient in many applications, airway resistance measured under such an approximation does not provide a useful parameter in predicting patient response to He/O_2_[12], as viscous and density-dependent resistances are lumped into the same term. The resistive loss coefficient used to quantify density-dependent airway resistance in the present study was a strong predictor of the reduction in inspiratory effort afforded by He/O_2_. It is our hope that such results will encourage clinicians working with He/O_2_ to re-examine and improve upon early techniques used to separate and quantify the different components of airway resistance, towards a goal of better identifying those patients that will respond to He/O_2_ therapy.

## Conclusions

The bench experiments presented above support the use of He/O_2_ to lower the inspiratory effort of patients with obstructive lung disease, provided that airway resistance includes a significant inertial, density-dependant component. Whereas pressure support lowered inspiratory effort by performing a portion of the total work of breathing for the simulated patient, He/O_2_ lowered inspiratory effort by reducing the contribution of resistive work to the total work of breathing. As compared to pressure support, the relative effect of breathing He/O_2_ to reduce inspiratory effort was strongly dependent on the severity and type of obstruction. When used in combination, reductions in inspiratory effort provided by He/O_2_ administration and pressure support were additive. Furthermore, in circumstances where pressure support NIV is not available or poorly tolerated, breathing He/O_2_ alone may provide a clinically significant reduction in inspiratory effort for some patients. Accordingly, efforts to better identify patients that will respond to He/O_2_ are well-warranted.

## Competing interests

AM, IK, GC, and JT are current employees of Air Liquide. KJ was employed by Air Liquide at the time of the study. LB is one of the principal investigators for an ongoing randomized controlled trial on the use of helium-oxygen during noninvasive ventilation and supported by Air Liquide.

## Authors’ contributions

AM conceived of the study, designed the experiments, participated in the collection and analysis of data, and drafted the manuscript; IK participated in the experimental design and analysis of data, and helped to draft the manuscript; KJ participated in the collection and analysis of data; GC contributed to the design of the experiments; LB and JT both contributed to the design of the experiments and analysis of data, and reviewed earlier versions of the manuscript. All authors read and approved the final manuscript.

## Pre-publication history

The pre-publication history for this paper can be accessed here:

http://www.biomedcentral.com/1471-2466/12/62/prepub
